# Determination of Optimal Reinforcement Ratios for Injection Molded Engineering Components: A Numerical Simulation

**DOI:** 10.3390/polym17202793

**Published:** 2025-10-19

**Authors:** Fuat Tan, Oğuz Veli Satı

**Affiliations:** Department of Mechanical Engineering, Balikesir University, 10145 Balikesir, Turkey; oguzvelisati@gmail.com

**Keywords:** glass fiber, injection molding, PA6, shrinkage, warpage

## Abstract

In this work, the influence of glass fibers on the performance of the injection molding process for a PA6-based AR15/M4 grip was investigated numerically. The process was realistically modeled using Autodesk Moldflow Insight for different glass fiber percentages (0 wt%, 15 wt%, 30 wt%, 45 wt%). The simulation results were evaluated, including the temperature distribution, flow time, pressure drop, pumping power, volumetric shrinkage and warpage displacement. The findings indicate that, with 15 wt% glass fibers, the material exhibits the shortest fill period (0.62 s) and the lowest pressure drop (0.0061 MPa) and power consumption (0.000433 kW), indicating maximum flow efficiency. On the other hand, a 30 wt% GF setup exhibited the largest volumetric shrinkage (17.76% at most) and warpage (Y: 1.213 mm), even though it had better thermal conductivity. The 45 wt% GF material exhibited the lowest amount of shrinkage and distortion but led to a greater energy consumption compared to 30 wt% GF. Overall, the 15 wt% GF grade provided the highest average process efficiency and dimensional accuracy; therefore, it is the most appropriate grade for precision molded firearm components.

## 1. Introduction

In recent years, glass fiber-reinforced thermoplastic composites have found increasingly widespread applications in the fields of engineering and construction owing to their thermal stability, high specific strength, and favorable moldability. In particular, polyamide 6 (PA6)-based systems stand out in the automotive, defense, and consumer product sectors due to their volumetric accuracy and processing flexibility. As a result, the popularity of these materials has risen significantly in these industries. Nevertheless, in injection molding, the control of manufacturing processes for parts with complex geometries, such as structural shells, connectors, and firearm grips, remains a challenging engineering problem, despite them offering considerable design flexibility.

PA6 occupies an important position among thermoplastics for engineering due to its high specific strength, good wear resistance, and chemical stability; however, neat PA6 suffers from certain drawbacks such as a high moisture absorption capacity and limited stiffness. To overcome these limitations, glass fiber reinforcement is commonly employed, which significantly enhances the elastic modulus, tensile strength, and dimensional stability. As a result, glass fiber-reinforced PA6 has become the preferred material, particularly for components with complex geometries that are used in the automotive, electronics, and defense industries [1,2,3]. Budiyantoro, C. et al. concluded in their study that polyamide 6 composites are advanced materials widely utilized for their lightweight and high-strength properties [4].

Injection molding is a fundamental manufacturing method based on melting thermoplastics, injecting them into a mold cavity under high pressure, and solidifying them through cooling. It offers advantages such as high reproducibility and the ability to produce complex geometries in a single step. However, its limitations include high mold costs, strong sensitivity to processing parameters, and the risk of filling or bonding defects. Today, the process extends beyond single-material parts to advanced applications such as overmolding (sequential molding of different materials), insert molding (embedding metallic or dissimilar materials), and overprinting using 3D printing (functionalizing surfaces through additive manufacturing) [5,6,7]. In this context, the properties of the final product are directly related to the distribution of fillers/reinforcements and the flow conditions; therefore, understanding the relationships among processing conditions, material structure, and final properties is critical from both an academic and an industrial perspective [8]. Angulo et al. reported that the mechanical performance of composites varies significantly depending on the processing methods employed and the fiber content. These effects lead to a notable increase in tensile strength, highlighting the critical importance of the manufacturing process and material composition in the performance of composite materials [9].

In the injection molding of PA6/CF composites, the reinforcement materials used substantially enhance the mechanical performance through increasing the degree of crystallinity and improving the viscoelastic properties. In this context, Herrmann et al. demonstrated in their study that accurately predicting the fiber orientation plays a crucial role in understanding the mechanical behavior of the material and in optimizing the molding process [10]. Juhász et al. investigated the influence of carbon-based nanoparticles in hybrid composite systems and demonstrated that the incorporation of basalt fibers and carbon additives enhances their interaction with the matrix phase, thereby providing superior mechanical properties [11]. Similarly, in a study conducted by Li et al., it was reported that PP/PTFE composite foams produced via microcellular injection molding exhibited notable improvements in both surface appearance and impact resistance during mold opening [12]. These findings support the influence of highly oriented structures on the mechanical performance. Wu et al. examined the distribution of carbon fibers in the injection weld line region, as well as the reinforcement effect at the interface, emphasizing that a uniform fiber distribution and high orientation significantly enhance the structural integrity, particularly in PA6 composites [13].

**Figure 1:**
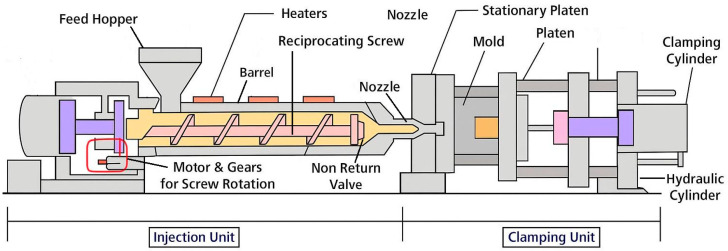
Injection molding machine units.

Considering these findings, it is essential to understand not only the material behavior but also the configuration of the injection molding equipment used in such processes. The general layout and functional units of the injection molding machine used in the present study are shown in Figure 1, serving as the basis for the simulations and analyses described in the following sections.

In composite materials produced via injection molding, the fiber orientation plays a decisive role in mechanical performance. Zhan et al. revealed that the flexural behavior of short glass fiber-reinforced PEEK composites is directly related to the fiber orientation [14]. Hirsch et al. validated the structural behavior observed during the injection molding process of thermoplastic composites reinforced with short and continuous fibers through both numerical simulations and experimental data. Their results showed that the orientation of short fibers has a direct influence on the mechanical properties of the final product [15]. In a study conducted by Karl et al., the flow–fiber coupling effect during mold filling was examined in detail, and it was determined that this interaction could lead to variations of up to ±30% in orientation ratios and of more than ±10% in stress fields [16]. Dogossy et al. demonstrated that the fiber orientation in thick-walled composite parts can be predicted with high accuracy using finite element analysis [17]. Chauhan V. et al. investigated the effects of different reinforcement ratios on the mold filling process using Moldflow simulations. By simulating various composite formulations, they compared the influence of fiber content on void formation and filling times, concluding that a fiber content of 10 wt% provided the most balanced filling process [18].

Numerous studies have examined the formation of weld lines in glass fiber-reinforced and unreinforced PA6 composites produced by injection molding, as well as their impact on mechanical properties. Li et al. reported that weld lines create microstructural weaknesses that reduce the material strength and that various methods have been developed to minimize these defects [19]. Mokarizadehhaghighishirazi et al. demonstrated that short glass fibers cause orientation losses in the weld line region, thereby reducing the mechanical strength [20]. Mrzljak et al., in their study based on the constant temperature approach, highlighted the effects of injection parameters on fatigue behavior [21]. Rochardjo and Budiyantoro investigated the role of the material structure and processing parameters on the wear resistance of reinforced PA6 hybrid composites [22], while Uyen et al. revealed that the packing pressure and melt temperature play a critical role in determining tensile strength through ANN-based analyses [23].

**Figure 2:**
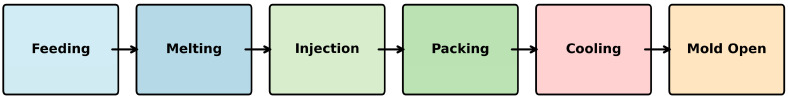
Injection molding process schematic.

On the process side, Nguyen et al. reported that LCI data do not sufficiently capture process variability, which introduces uncertainty into energy consumption analyses [24]. Zhao et al. emphasized that the measurement techniques developed for assessing machine condition, melt flow behavior, and product quality can provide a clearer understanding of the variabilities encountered during the molding process and enable optimization of the production process [25]. In a study by Dağlı et al., it was observed that mold shrinkage decreased from 1.29% to 0.7% with an increasing glass fiber content, while warpage was reduced to levels as low as 0.1 mm. In particular, polypropylene (PP) samples containing 20 wt% glass fiber exhibited high performance in ring flexibility tests, withstanding an up to 40% expansion in their inner diameter without fracture and offering the most suitable locking ring configuration [26]. In the work of Liu et al., it was found that the addition of PA6 strengthens the interfacial bonding between polymer and metal, and thereby a higher stress is required to detach molecular chains from the surface, ultimately providing a more robust joint [27]. Veneziani stated in his study that the Injection Molding Technique (IMT) enables the use of fluid and heated composites in a multilayer approach, thereby improving both esthetic and functional outcomes. This is analogous to the optimization of material properties through material distribution and layering in polymer composite applications [28]. Karthikeyan et al. reported that in composite materials reinforced with natural fibers and nano-SiC, the uniform dispersion of the reinforcement materials and their effective interaction with the matrix lead to significant improvements in mechanical properties, resulting in superior strength values [29]. Zhao et al. highlighted the critical role of quality control in minimizing shrinkage and warpage deformations, whereas Guerra et al. demonstrated the significant influence of the post-molding conditions and processing parameters on shape distortions in complex geometrical parts [30,31]. To contextualize the process flow in this study, a schematic representation of the injection molding process is shown in Figure 2.

Detailed examinations of the influence of processing parameters are supported by the work of Wilczyński et al., who demonstrated the effects of variables such as screw speed, plastication stroke, and back pressure on melting and flow behavior, as well as that by Dekel et al., who reported that high injection speeds combined with elevated mold temperatures lead to mechanical deterioration, whereas low speeds and lower mold temperatures result in performance improvements [32,33]. Baum et al. stressed the necessity of developing accurate simulation techniques by considering the interaction of geometric parameters that influence the dynamic evolution of fluids during the filling stage of the injection molding process [34]. Wang et al. stated that injection molding is a complex process used in the production of polymer composite products and identified the optimal processing conditions for improving the mechanical properties of PP SPCs as a barrel temperature of 260 °C, an injection pressure of 127.6 MPa, an injection speed of 0.18 m/s, and a holding time of 60 s [35]. Kuo et al. evaluated the cooling performance of injection molds produced by direct tooling (DT) and indirect tooling (IDT) methods, identifying the most suitable method for each case based on total production cost, cooling time, and bending strength [36]. Fu et al. noted that injection molding is widely applied across numerous industries from consumer products to aerospace components and that it is continuing to evolve in response to new material processing challenges and technological advancements [37]. Li et al. stated that, in the injection molding process of short fiber-reinforced composites, warpage, shrinkage, and residual stresses are critical quality criteria. They concluded that these parameters are significantly influenced by processing conditions and fiber properties. Moldflow simulations and optimization methods are an effective approach for improving quality [38]. Amjadi et al. proposed a critical plane-based fatigue damage model for predicting the tension–tension or tension–compression fatigue life of short glass fiber-reinforced thermoplastics, taking into account the effects of fiber orientation [39].

In addition, Lee et al. elaborated on the effects of weld lines on the fracture behavior and mechanical properties of glass fiber-reinforced and unreinforced PA6 produced by injection molding [40]. Ucpinar et al. reported that short glass fiber-reinforced composites were produced using the melt blending method with a twin-screw extruder, followed by injection molding [41]. Artykbaeva et al. observed that increasing the glass fiber content in PA6/PA610 blends reduced the water absorption (WA) value of neat PA6, demonstrating the influence of glass fibers on the properties of PA6/PA610 composites [42]. More recently, studies conducted by Tan and colleagues have made significant contributions to understanding the behavior of polymers under different material and process conditions in injection molding. For instance, using the finite element method (FEM), Tan et al. obtained the optimum material and process parameters for piezoresistive card-type pressure sensors [43]; Tan and Alkan investigated the influence of cooling parameters on melt behavior during microinjection molding [44]; Tan optimized the mechanical properties of PA66+PA6I/6T composites by employing Response Surface Methodology (RSM) combined with the Grey Wolf approach [45]; and Tan and Alkan numerically analyzed the thermophysical and mechanical performance of an injection-molded femur implant [46].

Previous studies have primarily focused on individual outputs (such as shrinkage, warpage, or energy), while systematic investigations addressing the multidimensional effects of the glass fiber content in complex, defense-oriented components under identical boundary conditions remain limited. The present study aims to fill this gap by conducting a detailed and comparative analysis of the injection molding behavior of a firearm grip made of PA6 with four different glass fiber reinforcement levels (0, 15, 30, and 45 wt%) using Autodesk Moldflow Insight. The analysis is not limited to shrinkage and warpage but also encompasses key outputs such as the temperature distribution, pressure drop, flow characteristics, and energy input. The findings quantitatively reveal the complex balance between reinforcement content, process efficiency, and dimensional stability, thereby providing an engineering-based reference for material and process optimization in high-performance polymer applications.

## 2. Materials and Methods

### 2.1. Material

The polymer employed in this study is based on polyamide 6 (PA6) thermoplastics. Four different commercial grades were considered in the simulation experiments, including both non-reinforced systems and systems with various levels of glass fiber reinforcement: Novamid 1015 (0 wt% glass fiber), Novamid 1015G15 (15 wt% glass fiber), Novamid 1015G30 (30 wt% glass fiber), and Novamid 1015G45 (45 wt% glass fiber). All materials were supplied by DSM Engineering Materials (Heerlen, Netherlands); the fundamental physical and mechanical properties of these materials are summarized in Table 1. These values were obtained from the technical datasheets and were used as input parameters in the simulations.

### 2.2. Model and Mesh

**Figure 3:**
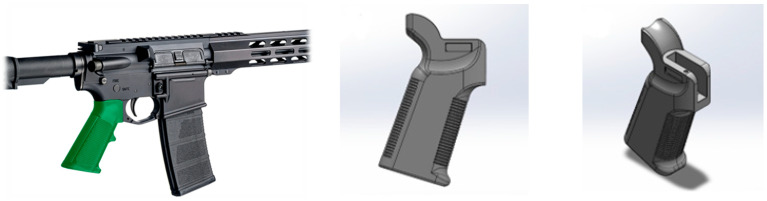
AR15/M4 Rifle model and manufacturing product views.

The component analyzed in this study is the grip of the AR15/M4 rifle, a critical interface between the user and the rifle. This part features a combination of ribbed textures for improved grip, curved ergonomic contours, and a mounting structure designed to connect securely with the lower receiver. Its geometry includes thin-walled regions, sharp transitions, and long unsupported surfaces, all of which make it highly susceptible to warpage and shrinkage during injection molding. Due to its direct contact with the user’s hand and its mechanical role in firearm stability, high dimensional accuracy, surface quality, and structural integrity are essential in this component, making it an ideal subject for reinforcement-dependent process simulation. Visuals of the 3D model of this part are shown in Figure 3.

**Figure 4:**
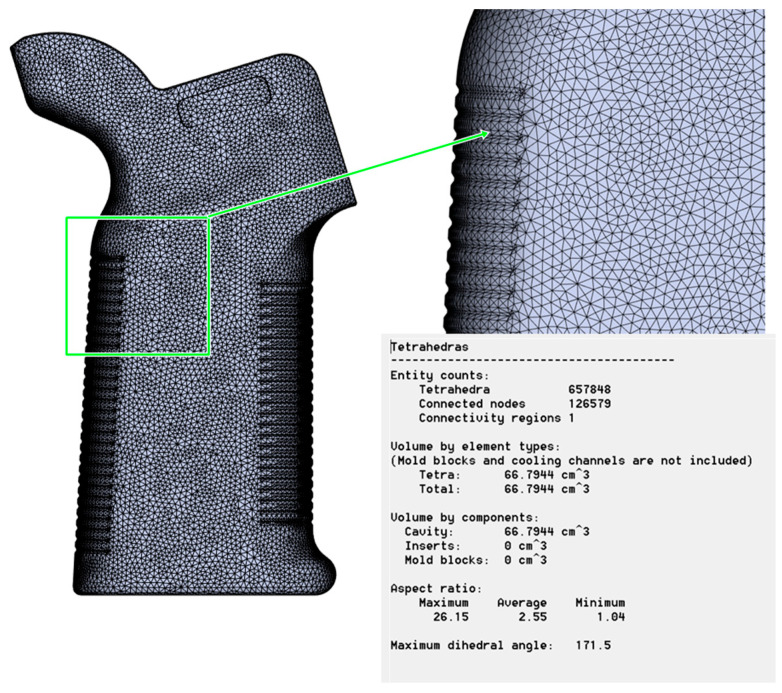
Mesh model and metrics.

All models featured three-dimensional tetrahedral elements of the best mesh quality, as can be seen in Figure 4. The mesh consisted of about 657,848 tetrahedral elements and 126,579 nodes to capture the complexity of the AR15/M4 pistol grip, particularly its ribbed and curved nature. The total mesh volume was 66.79 cm^3^, equivalent to the cavity volume, and cooling and mold inserts were excluded. Mesh quality metrics were carefully monitored throughout the simulation to ensure that it remained stable and converged. The highest aspect ratio was 26.15, whereas the average and lowest were 2.25 and 1.04, respectively. These values indicate sufficient refinement and element uniformity across the domain. The maximum dihedral angle recorded was 171.5°, remaining within the acceptable limits for accurate finite volume computation. The magnified section of the mesh (top right, Figure 4) highlights the local refinement in thin walled regions, where thermal gradients and shrinkage effects are expected to be most pronounced.

### 2.3. Process and Machine Setup

All simulations were performed using Autodesk Moldflow Insight 2016, applying identical process conditions across all material configurations to ensure that the observed differences could be attributed solely to the variation in glass fiber content. The selected process parameters were based on industry-relevant settings for high-performance polyamide components with complex geometries. The melt temperature was fixed at 270 °C, while the mold surface temperature ranged between 81.82 °C (30 wt% GF) and 83.33 °C (0 wt% GF). A uniform cooling time of 20.0 s was used to ensure comparability of heat removal performance between reinforcement levels. The maximum injection pressure was set to 180 MPa and the screw intensification ratio was defined as 10.0, yielding a realistic pressure amplification in the simulation. The clamp force was modeled with a capacity of 1.0194 × 10^33^ N, exceeding the theoretical requirements to prevent mold separation during the injection cycle.

The filling phase was configured to be flow rate-controlled. Due to differences in viscosity and thermal conductivity, the fill time varied among materials: 0.62 s for 15 wt% and 30 wt% GF and 1.13 s for 0 wt% and 45 wt% GF. The velocity-to-pressure switch-over was activated at 100% volume in all cases; however, the switch-over pressure ranged from 30.92 MPa (30 wt% GF) to 47.69 MPa (15 wt% GF), indicating material-dependent resistance to flow during cavity filling. A packing pressure was applied through a two-stage profile with both hold and decay steps, and the total injection hold time was maintained at approximately 30 s for each case. The cooling performance and flow resistance were further analyzed through the frozen volume ratio, Reynolds number, pressure drop, and pumping power outputs, extracted directly from the results. This standardized setup enabled a controlled comparison of how the reinforcement ratio affects thermal distribution, shrinkage behavior, and energy demand in the injection molding of geometrically demanding components such as firearm grips. All simulations were carried out under identical processing conditions for the different glass fiber contents. The parameters are summarized in Table 2.

### 2.4. Gate Location Design

**Figure 5:**
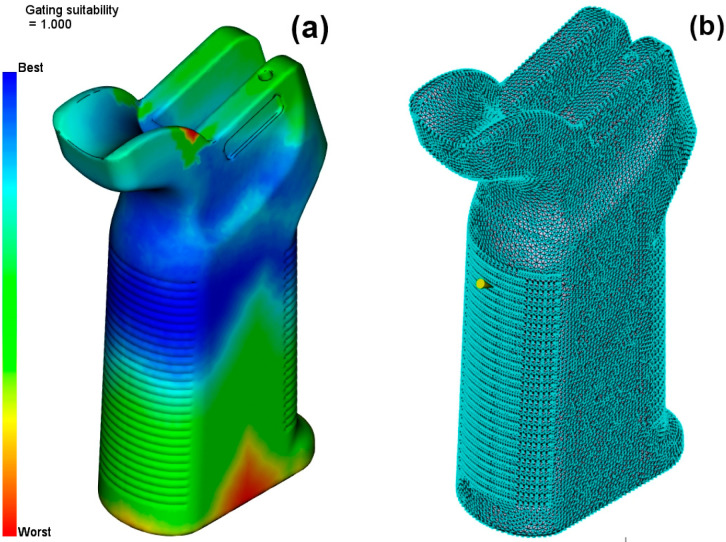
(**a**) Gating suitability. (**b**) Injection location.

In the next step, Gate Location analysis was conducted for the model. This analysis is a critical step for the model to be filled correctly. The right injection entry point chosen leads to a balanced distribution of the fluid within the part and prevents flow problems. The optimized entry point indicated in Figure 5 was specified to be the model’s balanced filling point; thus, the efficiency of the production process also increased. This phase is very important in increasing part quality and, at the same time, minimizing production faults.

### 2.5. Cooling Circuit and Runner System Design

**Figure 6:**
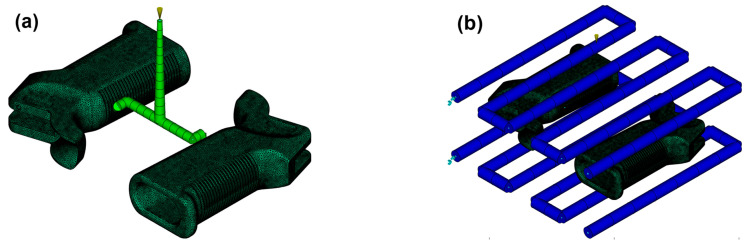
(**a**) Cooling system. (**b**) Runner system.

Another crucial phase of this project is the research on cooling channels and runner systems. The cooling channels were meticulously and optimally designed via an analysis of part behavior using the Cool–Fill–Pack–Warp method. The efficiency of the cooling process allows the uniform and efficient distribution of the fluid within the mold. The design that is shown in Figure 6 accounts for the placement of the runner such that its structural parts can also be reached accordingly. The mold was assumed to be manufactured from P20 tool steel, which is commonly employed in industrial injection mold production owing to its good machinability, polishability, and balanced strength–toughness properties. In the present study, the mold was not physically fabricated; instead, the cooling circuit and runner system were virtually modeled in Autodesk Moldflow Insight to replicate realistic industrial practice. This approach allows accurate evaluation of thermal management and flow distribution without the need for costly tooling.

The fundamental geometrical parameters of the system are summarized in Table 3. The injection mold was made in such a way that it has a three-channel cooling system with a diameter of 10 mm and an overlaying distance of as high as 30 mm, with a distance of 80 mm on the surface, to ensure uniform cooling across the grip. The runner system was designed with a circular runner with a diameter of 6 mm, the entry point of which was a 3 mm sprue hole with a 110 mm length. The output holes were 3 mm in diameter, and those on the side were 8.49 mm long. The parting plane was at Z = −6.56 mm, with the position of the entry prong placed off-center, which could likely have an impact on the filling process. The layout of this mold was designed such that cooling would be fast and effective and so that the pressure loss in the important areas of the grip would be at a minimum in the design of the firearm. This was accomplished by taking the right design path.

## 3. Results

### 3.1. Temperature Distribution

**Figure 7:**
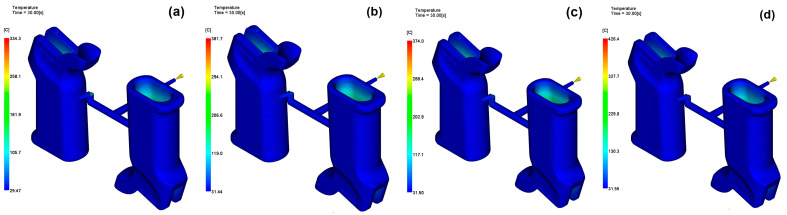
Temperature distribution for (**a**) 0 wt%, (**b**) 15 wt%, (**c**) 30 wt%, (**d**) 45 wt%.

All four of the different glass fiber reinforcement ratios were compared with regard to the temperature distribution, and thus the heat removal performance, under the same process conditions. According to the simulation results, the higher the reinforcement ratio, the lower the part surface temperature and the average external temperature of the mold. The highest part surface temperature of 188.93 °C was recorded in the unfilled PA6 and decreased to 186.72 °C at the 30 wt% reinforcement level; the minimum values were similar. Such findings indicate that the fiber-reinforced materials allow for more efficient heat transfer during the cooling stage due to their higher thermal conductivity. The temperature distribution under identical process conditions is shown in Figure 7.

The same situation was observed in the average molded outer surface temperature; that is, it dropped slightly just after the addition of fibers and reached the lowest level at 30 wt% addition. However, a slight rise at 45 wt% means that very-high-fiber parts can reduce the uniformity of the melt flow and thus a non-uniform heat distribution will occur, leading to heat being retained in those areas only. Similarly, the extent of heat removal followed this trend, with 15 wt% and 30 wt% GF grades removing heat more effectively than unfilled or 45 wt% configurations. A combination of augmented thermal conductivity and thus a lower melt viscosity is to be credited for the superior thermal behavior, as this aids in the increasing the speed of the solidification process.

In fact, despite the better cooling performance, the increased reinforcement ratios do not appear to have an impact on warpage or shrinkage reductions; this will be discussed in later sections. This underlines the complicated interplay between thermal responses and mechanical deformation. In general, the findings validate that moderate reinforcement levels (15–30 wt%) lead to a superlative equilibrium between heat sinking and process steadiness; thus, they are more useful for the high-precision molding of thin-walled and ribbed structures in firearm components.

### 3.2. Flow Behavior and Pressure Characteristics

In this study, the influence of the different glass fiber contents on the flow properties of PA6 was determined on the basis of some important parameters like the pressure drop, pumping power, fill time and velocity-to-pressure (V/P) switch-over pressure. When the reinforcement ratio increased, there was a non-linear trend in flow resistance. The lowest pressure drop was recorded for the 15 wt% reinforced material (0.0061 MPa), followed by a slight increase at 30 wt% and 45 wt%. This highlights that moderate reinforcement improves flowability, probably due to the enhancement in thermal conductivity and the reduction in milling viscosity during the initial filling process. However, in the case of a high fiber content, the melt stiffness increases, leading to resistance to the flow and a small increase in the pressure loss.

The energy required to keep the flow rate steady was related to the trend in pressure drop. The maximum flow efficiency was observed for the 15 wt% GF configuration (0.000433 kW), which was the minimum pumping power required. The addition of unfilled material, along with the 45 wt% GF material, required a slightly higher energy input, which in turn indicated the possibility of polymer fiber interactions, resulting in either a greater viscosity or shear stress. With respect to the filling dynamics of the various models, the filling time for the 15 wt% and 30 wt% reinforced models was 0.62 s, noticeably shorter than that for the unfilled and 45 wt% models (1.13 s).

**Figure 8:**
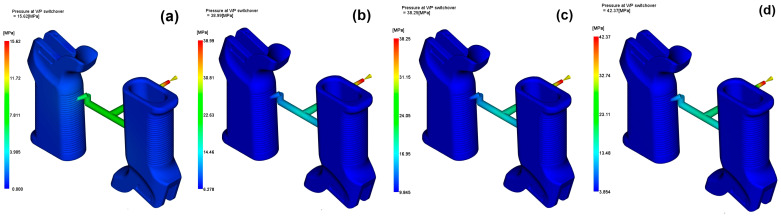
Pressure at V/P switch-over for (**a**) 0 wt%, (**b**) 15 wt%, (**c**) 30 wt%, (**d**) 45 wt%.

This finding demonstrates a good balance between the flow features and the mid-range reinforcement levels, where the melting remains sufficiently active without too high a stiffness or undershearing occurring. The variation in pressure at the velocity-to-pressure switch-over point for each reinforcement level is illustrated in Figure 8. The V/P switch-over pressures exhibited substantial differences. The 15 wt% GF material demonstrated the greatest transition pressure (47.69 MPa), implying that the cavity was filled to the brim at the switching phase.

On the other hand, the 30 wt% GF material exhibited the lowest pressure (30.92 MPa), which might reflect an earlier switch than intended or easier cavity saturation owing to better heat transfer. The overall results show that 15 wt% of glass fibers achieves the best balance between flow efficiency and energy consumption, but excessive reinforcement may make the flow resistant, which in part undermines the thermal benefits. These results are of high importance for firearm components with long, thin or ribbed geometries, for which uniform filling and energy efficiency are the most important factors.

### 3.3. Volumetric Shrinkage

**Figure 9:**
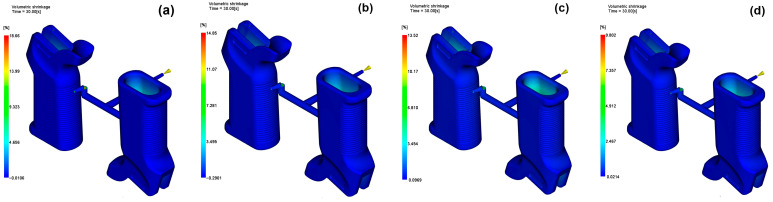
Volumetric shrinkage for (**a**) 0 wt%, (**b**) 15 wt%, (**c**) 30 wt%, (**d**) 45 wt%.

Volumetric shrinkage is a key aspect to consider in injection molding, particularly for components that require structural integrity and have intricate geometries like the AR15/M4 firearm grip. This study compares the maximum, average and root mean square shrinkage values for four reinforcement percentages (0 wt%, 15 wt%, 30 wt% and 45 wt%), as well as the dimensional stability of the system for each material configuration. It was found through simulation analysis that volumetric shrinkage increased with the glass fiber content, but they were not correlated in a linear way. The highest volume shrinkage of 17.76% was observed at the 30 wt% GF configuration, while the unfilled PA6 had only a maximum shrinkage of 13.57%, which was the lowest. This can be attributed to the accumulation of internal stress and the uneven packing efficiency in mid-range reinforced composite materials. The average shrinkage was also highest at a fiber grade of 30 wt% (12.18%), with the other configurations of 15 wt% and 45 wt% exhibiting approximately the same mean values of around 10.5%. The corresponding volumetric shrinkage distributions for the four reinforcement levels are presented in Figure 9.

It is demonstrated by these results that fiber reinforcement primarily enhances dimensional predictability, but intermediate levels of reinforcement (such as 30 wt%) can also cause inconvenient local shrinkage gradients. Therefore, both the type of material and the method used to fill the medium must be determined so that shrinkage-related distortions are minimized in firearm components. A similar trend was reported by Artykbaeva [42], who observed that, at mid-range reinforcement levels of PA6 composites, internal shrinkage increased due to uneven packing stress and rapid solidification.

### 3.4. Warpage

Warpage, which is characterized as the unwanted deviation of a molded part from its intended dimensions due to irregular shrinkage and residual stress, is a prominent problem in the field of firearm grip components, as they must be ergonomically precise and exhibit structural integrity. This study evaluated warpage effects using the maximum displacement data in the X, Y and Z directional dimensions at all four glass fiber reinforcement levels. The simulation results suggest that the assumption that the higher the reinforcement, the lower the warpage is not accurate. In the 30 wt% GF model, which had the highest total deformation, the maximum Y-direction displacement was 1.213 mm. This indicates that 30 wt% GF, while satisfying the requirements for thermal conductivity and cycle time reductions, leads to higher rates of residual stresses due to fast cooling and the lack of molecular relaxation.

**Figure 10:**
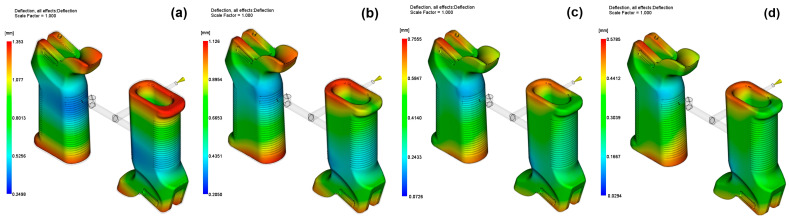
Warpage for (**a**) 0 wt%, (**b**) 15 wt%, (**c**) 30 wt%, (**d**) 45 wt%.

On the contrary, the configurations of 0 wt% and 45 wt% GF had lower displacement values, while the 45 wt% model produced warpage results which were equal to or better than the unfilled PA6. This behavior shows the inconsistency of the fiber content and warpage, which is probably a result of a shift from augmentation in stiffness to the blockage of flow-induced orientation during solidification. The simulated warpage patterns at different reinforcement ratios are shown in Figure 10.

In addition, analysis of the directional data indicated that the displacement of the X and Z axes was relatively low. It was then inferred that the Y-direction, which is aligned with the long axis of the grip, is the most sensitive to the effects of the fiber. This is in line with the geometry of the part, where long-axis behavior and uneven cooling are the main features of the deformation. The outcomes are consistent with those of the research carried out by Lee [40], who pointed out that the deformation in the Y-direction generally dominates in long-axis polymer materials, particularly when the cooling conditions are uneven.

## 4. Discussion

Shrinkage and warpage behaviors in short glass-fiber-reinforced polyamides are governed by the fiber orientation, interfacial interactions, and crystallization processes. Analyses revealed that, as the fiber content increased, the average shrinkage decreased from 12.0% (0 wt%) to 10.5% (45 wt%), while warpage was reduced from 1.27 mm to 0.53 mm. At 30 wt% GF; however, the maximum warpage reached 1.213 mm, with an RMS warpage value of 0.67 mm. This trend is consistent with classical orientation models [47], which demonstrate that fiber alignment in the flow direction during mold filling induces anisotropic deformation.

Interfacial interactions were also decisive in shaping the results. Although fiber– matrix bonding enhances load transfer with increasing reinforcement, it may simultaneously generate localized stress concentrations at the fiber ends, thereby contributing to greater warpage tendencies. This explains the relatively high warpage observed at 30 wt% GF, where the maximum Y-direction warpage reached 1.213 mm, while the RMS warpage value was 0.67 mm. Thomason demonstrated that the interfacial properties in glass fiber-reinforced polyamides directly influence mechanical and dimensional behavior [1], while Dogossy et al. reported that Moldflow predictions for shrinkage showed deviations of less than 5% from experimental values, in agreement with the present results [17].

The crystallization process further clarifies the dimensional stability at higher reinforcement levels. The nucleating effect of glass fibers accelerates crystallization, restricting chain mobility and reducing volumetric changes. This effect was confirmed in the 45 wt% GF case, where the average shrinkage dropped to 10.5%, while the RMS shrinkage was 5.5%. Takagi et al. experimentally demonstrated that fibers accelerate crystallization in PA6 and reduce shrinkage, while Lee et al. reported that crystallization-induced anisotropy strongly affects the mechanical responses of short fiber-reinforced PA6 composites [40]. Thus, the obtained results not only validate macroscopic deformation trends but also confirm the microstructural mechanisms described in the literature, providing an explanatory rather than merely descriptive framework.

One limitation of this study is the lack of experimental validation. However, the numerical trends observed are highly consistent with experimental studies on PA6-GF systems. Ryu et al. experimentally reported an average shrinkage of 12–13% and a maximum warpage of approximately 1.2 mm in 30 wt% GF-PA6 parts and demonstrated that Moldflow simulations could predict these values within a 10% error margin [48]. In the present study, for 30 wt% GF, the average shrinkage was found to be 12.18% and the maximum warpage along the Y-direction was 1.213 mm, showing close agreement with the above results. Similarly, they presented experimental measurements of warpage resistance and dimensional stability in 30 wt% GF-PA6 specimens, with Moldflow predictions confirmed within a 5–8% deviation. Furthermore, Dogossy et al. experimentally demonstrated that Moldflow predicted the shrinkage in thick-walled PA6-GF parts within a 5% error margin [17]. These findings indicate that the numerical results obtained in this study are not only theoretically reliable but also supported by direct experimental validations reported for PA6-GF composites. Therefore, although the absence of experimental testing remains a limitation, the proven validity of the applied models and the close agreement with experimental data in the literature reinforce the reliability of the present study. To provide a clearer comparison of the processing and structural responses, the key simulation results at different glass fiber ratios are summarized in Table 4.

The functional performance of a firearm grip is not solely determined by dimensional stability; parameters such as the stiffness/load-bearing capacity (E, G), impact/energy absorption, and fatigue life are equally critical. Although no mechanical tests were performed in this study, classical theories for short glass-fiber-reinforced PA6, as well as material–process log outputs, allow for quantitative predictions of these parameters. In short fiber composites, the effective elastic modulus increases with the fiber volume fraction (φ) and orientation factor (η_0_), while orientation-induced anisotropy governs dimensional deviations [47]. For the PA6 grades used in this work, the elastic modulus increased from 2910 MPa (0 wt%) to 12,750 MPa (45 wt%) (≈4.38×), while the shear modulus rose from 1050 MPa to 3189 MPa (≈3.04×) (Table 1). Such increases are expected to reduce deformation amplitudes under load, thereby enhancing the load-bearing capacity and fatigue resistance, while simultaneously amplifying orientation-induced anisotropy [47]; Thomason, Consistent with Rosen–Hashin-type mixture theories, the coefficient of thermal expansion (CTE) decreases with reinforcement [1]. According to the log-file data, the CTE dropped from 8.15 × 10^−5^ 1/°C (0 wt%) to 1.885 × 10^−5^ 1/°C (45 wt%), corresponding to a ≈77% reduction (Table 1). This decrease minimizes thermomechanical stress during thermal cycling, favorably influencing the dimensional stability and fatigue resistance. Furthermore, the nucleating role of glass fibers accelerates crystallization, reducing compressibility; this mechanism is corroborated by the observed reduction in average volumetric shrinkage from 12.00% (0 wt%) → 9.06% (15–30 wt%) → 5.47% (45 wt%) [40].

With increasing reinforcement, both orientation anisotropy and interfacial stress transfer intensify. Accordingly, local stress concentrations become more pronounced at 30 wt% GF (V/P switchover pressure ≈ 38.25 MPa; filling time 1.23 s), whereas at 45 wt% GF, the combined effects of a higher stiffness and a lower CTE tend to limit shrinkage and warpage (average shrinkage: 5.47%). On the other hand, processing demands also increase (V/P ≈ 42.37 MPa; filling time: 1.54 s; mold temperature: ~83 °C). These opposing effects highlight the need for the holistic optimization of the dimensional accuracy–stiffness–fatigue resistance triad in industrial firearm grip design [1].

## 5. Conclusions

The present article features a thorough numerical analysis concerning the effects of changing the glass fiber reinforcement ratios (0 wt%, 15 wt%, 30 wt%, 45 wt%) in the injection molding process of a very difficult-to-mold component of the PA6 firearm grip. This study attempted to determine the optimum degree of reinforcement based on an analysis of 61 simulation outcomes, including thermal, flow, shrink, warpage, and energy parameters. Through this, it could be ascertained that the desired dimensional stability, process efficiency, and structural performance could be achieved.

The results of the experiment suggest that the influence of glass fiber reinforcements on the process of injection molding, in the case of PA6, has non-linear characteristics and multi-dimensionality. Even though the thermal conductivity and stiffness are enhanced by the increase in fiber content, these enhancements do not automatically guarantee a superior dimensional stability or low energy consumption. From the various configurations assessed, 15 wt% GF presented the best compromise between cooling efficiency, lower pressure loss, distortion, and energy conservation.

The 30 wt% GF composition was observed to exhibit the greatest volumetric shrinkage and warpage in spite of its advantageous thermal properties. The performance of the 45 wt% GF material, however, is on a level similar to PA6 without filler, but its energy expenditures are higher. The findings indicate that the best and most reasonable combination of mechanical performance and process efficiency can be obtained through mid-range reinforcement (15 wt%), and this is suitable for precision molded parts such as firearm grips.

The simulation data revealed that 45 wt% GF-PA6 offers dimensional advantages through reduced shrinkage and warpage values. However, this level of reinforcement requires more precise control of processing parameters. For instance, the prolonged packing pressure (33.9 MPa), the cooling system demand of approximately 0.6 kW for heat removal, and the temperature rise of ΔT ≈ 2.1 °C are aspects of the process that necessitate further optimization. The literature also reports that high fiber loadings (>40 wt%) increase the melt viscosity, thereby complicating mold filling and fiber distribution [16,40]. Therefore, for the practical realization of the advantages offered by the 45 wt% configuration in industrial applications, specialized runner designs, elevated injection pressures, and optimized cooling strategies are required.

In summary, it is necessary to choose a reinforcement ratio cautiously based on the specific performance goals of the application. As seen in the case of the firearm grips, where both ergonomic precision and mechanical durability are important, the 15 wt% GF configuration seems to provide an optimal balance. Further studies could include experimental confirmation of the findings and further optimization through shape and packing profile refinement. Despite this study offering a comprehensive numerical assessment of the influence of glass fiber strengthening on the injection molding behavior of pistol grip parts, additional studies are required to corroborate and expand the results. In addition, an analysis of fiber orientation regarding the gates and flow direction, in particular, may provide more information about the trends in stress distribution and deformation. An informed approach could be adopted in regulating dimensional stability by studying the effect of gate design, packing pressure profiles, and the use of multi-objective optimization techniques. Finally, performing fatigue and mechanical tests on molded parts would help achieve a more complete understanding of the structural effects of different levels of reinforcement in real-world scenarios.

## Figures and Tables

**Table 1:** Properties of materials.

DSM Japan Engineering Plastics	Novamid 1015G	Novamid 1015G15	Novamid 1015G30	Novamid 1015G45
Glass Fiber Ratio (wt%)	0	15	30	45
Elasticity Modulus (MPa)	2910	5390.1	8802.75	12,750.1
Poisson Ratio (υ)	0.386	0.4032	0.4057	0.4002
Shear Modulus (MPa)	1050	1554.91	2288.73	3189.61
Melt Density (g/cm^3^)	0.89912	1.0576	1.2035	1.3511
Thermal Expansion Coefficient (1/C)	8.15 × 10^−5^	4.524 × 10^−5^	2.758 × 10^−5^	1.885 × 10^−5^

**Table 2:** Process parameters and machine settings.

Parameter	Value/Range	Description
Melt temperature (°C)	270	Constant for all materials
Mold surface temperature (°C)	81.82 (30 wt% GF)–83.33 (0 wt% GF)	Slight variation with fiber content
Injection pressure (MPa)	Max. 180	Screw intensification ratio: 10.0
Clamp force (N)	1.0194 × 10^33^	Ensures mold closure under maximum load
Filling control	Flow rate-controlled	Same for all cases
Filling time (s)	0.62 (15–30 wt% GF)–1.13 (0–45 wt% GF)	Depends on viscosity and thermal conductivity
V/P switchover pressure (MPa)	30.92 (30 wt% GF)–47.69 (15 wt% GF)	Varies with material flow resistance
Packing pressure profile	Two-stage (holding + reduction)	Constant for all cases
Holding time (s)	30	Total duration of the packing phase
Cooling time (s)	20.0	Constant for all materials

**Table 3:** Cooling and runner system dimensions.

Cooling System Dimensions (mm)		Runner System Dimensions (mm)	
Part dimension (X)	167.11	Specify the sprue position (X)	−58.42
Part dimension (Y)	109.05	Specify the sprue position (Y)	−40.64
Part dimension (Z)	28.03	Parting plane (Z)	−6.56
Channel diameter	10	Sprue orifice diameter	3
Alignment shape of the circuit	25	Runners diameter	6
Number of channels per part	3	Sprue length	110
Distance between channel centers	30	Side gate orifice diameter	3
Distance from part edge	80	Side gate length	8.49
Top and bottom distance	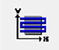	Include angle	3

**Table 4:** Summary of key simulation results for different glass fiber ratios.

GF Ratio (wt%)	Avg. Shrinkage (%)	Max. Warpage (mm)	CTE (×10^−5^ 1/°C)	Fill Time (s)
0	12.0	1.27	8.15	1.13
15	10.5	0.7	4.52	0.62
30	12.18	1.213	2.76	0.62
45	10.5	0.53	1.89	1.13

## Data Availability

The original contributions presented in this study are included in the article material. Further inquiries can be directed to the corresponding authors.

## Abbreviations

The following abbreviations are used in this manuscript:

GF	Glass Fiber
PVT	Pressure–Volume–Temperature
CF	Carbon Fiber
CTE	Coefficient of Thermal Expansion
IMT	Injection Molding Technique
IDT	Indirect Tooling
V/P	Velocity-to-Pressure (switchover)
RMS	Root Mean Square
LCI	Life Cycle Inventory

## References

[1] Thomason J. (2009). The influence of fibre length, diameter and concentration on the impact performance of long glass-fibre reinforced polyamide 6,6. Compos. Part A.

[2] Sanlı S., Durmus A., Ercan N. (2012). Isothermal Crystallization Kinetics of Glass Fiber and Mineral-Filled Polyamide 6 Composites. J. Mater. Sci..

[3] De Monte M., Moosbrugger E., Quaresimin M. (2010). Influence of Temperature and Thickness on the off-Axis Behaviour of Short Glass Fibre Reinforced Polyamide 6.6—Quasi-Static Loading. Compos. Part A Appl. Sci. Manuf..

[4] Budiyantoro C., Rochardjo H.S., Wicaksono S.E., Ad M.A., Saputra I.N., Alif R. (2024). Impact and Tensile Properties of Injection-Molded Glass Fiber Reinforced Polyamide 6–Processing Temperature and Pressure Optimization. Int. J. Technol..

[5] Michaeli W. (2014). Plastics Processing: An Introduction.

[6] Osswald T.A., Hernández-Ortiz J.P. (2006). Polymer Processing: Modeling and Simulation.

[7] Strong A.B. (1989). Fundamentals of Composites Manufacturing: Materials, Methods and Applications.

[8] Zhou S., Hrymak A.N. (2024). Injection Molding of Polymers & Polymer Composites. Polymers.

[9] Angulo C., Shackleford L., Ning H., Pillay S. (2024). Comparative study on the mechanical behaviors of compression molded, additively manufactured, and injection molded recycled carbon fiber reinforced rHDPE composites. Compos. Part B Eng..

[10] Herrmann T., Niedziela D., Salimova D., Schweiger T. (2024). Predicting the fiber orientation of injection molded components and the geometry influence with neural networks. J. Compos. Mater..

[11] Juhász Z., Pinke B., Gonda B., Mészáros L. (2024). Effects of carbon-based nanoparticles on the properties of poly (lactic acid) hybrid composites containing basalt fibers and carbon-based nanoparticles processed by injection molding. Polym. Eng. Sci..

[12] Li X., Zuo Z., Mi H.Y., Zhao P., Dong B., Liu C., Shen C. (2024). High-strength, impact-resistant PP/PTFE composite foam with enhanced surface appearance achieved through mold-opening microcellular injection molding. Polymer.

[13] Wu H., Qiu J., Zhang G., Sakai E., Zhao W., Tang J., Guo S. (2024). Distribution and reinforcement effect of carbon fiber at the interface in injection welding of polyamide 6 composites. Polym. Compos..

[14] Zhan Z., Wu W., Zhao J., Guo Y., Su D., Ge Y. (2024). A multiscale scheme for homogenization to characterize the flexural performances of injection molded short glass fiber reinforced polyether ether ketone composites. Polym. Compos..

[15] Hirsch P., John M., Leipold D., Henkel A., Gipser S., Schlimper R., Zscheyge M. (2021). Numerical simulation and experimental validation of hybrid injection molded short and continuous fiber-reinforced thermoplastic composites. Polymers.

[16] Karl T., Zartmann J., Dalpke S., Gatti D., Frohnapfel B., Böhlke T. (2023). Influence of flow–fiber coupling during mold-filling on the stress field in short-fiber reinforced composites. Comput. Mech..

[17] Dogossy G., Morauszki T., Ronkay F. (2022). Experimental investigation and applicability of multi-stage simulations in the case of a thick-walled injection-moulded composite. Appl. Sci..

[18] Chauhan V., Kärki T., Varis J. (2023). Process Simulation of Compression Molding Process and Effect of Fiber Content on Recycled Polymer Natural Fiber Composites Using Moldflow Analysis. Intelligent and Transformative Production in Pandemic Times.

[19] Li X.J., Zuo Z.M., Mi H.Y., Dong B.B., Antwi-Afari M.F., Liu C.T., Shen C.Y. (2024). A review of research progress on the minimization of weld lines in injection molding. Int. J. Adv. Manuf. Technol..

[20] Mokarizadehhaghighishirazi M., Buffel B., Lomov S.V., Desplentere F. (2024). Investigation of microstructural and mechanical properties of weld lines in injection-molded short glass fiber-reinforced polyamide 6. Polym. Compos..

[21] Mrzljak S., Delp A., Schlink A., Zarges J.C., Hülsbusch D., Heim H.P., Walther F. (2021). Constant temperature approach for the assessment of injection molding parameter influence on the fatigue behavior of short glass fiber reinforced polyamide 6. Polymers.

[22] Rochardjo H.S., Budiyantoro C. (2021). Manufacturing and analysis of overmolded hybrid fiber polyamide 6 composite. Polymers.

[23] Uyen T.M.T., Nguyen H.T., Nguyen V.T., Minh P.S., Do T.T., Nguyen V.T.T. (2024). Optimizing the Tensile Strength of Weld Lines in Glass Fiber Composite Injection Molding. Materials.

[24] Nguyen D.T., Yu E., Barry C., Chen W.T. (2024). Energy consumption variability in life cycle assessments of injection molding processes: A critical review and future outlooks. J. Clean. Prod..

[25] Zhao N.Y., Liu J.F., Su M.Y., Xu Z.B. (2024). Measurement techniques in injection molding: A comprehensive review of machine status detection, molten resin flow state characterization, and component quality adjustment. Measurement.

[26] Dağlı M., Demirer A., Yumat E. (2025). Effect of glass fiber reinforcement on compressive flexibility and dimensional stability in injection-molded polypropylene composite locking ring. Pamukkale Üniversitesi Mühendislik Bilim. Derg..

[27] Liu D., Liu S., Zhang S., Evsyukov S.A., Luo X. (2025). Study on the interfacial bonding mechanism of nano-injection molding PPS/PA6@ Al: A molecular dynamics simulation study. Polym. Eng. Sci..

[28] Veneziani M. (2025). Composite Injection Molding. Int. J. Esthet. Dent..

[29] Karthikeyan M.K.V., Kamaraj L., Kavipriya S., Rathinavelu V., Sadagopan D.K., Soudagar M.E.M., Manickaraj P. (2024). Investigation and chemical processing effect of sisal fiber epoxy composite characteristic enhancement with nano-SiC via injection mold. Int. J. Adv. Manuf. Technol..

[30] Zhao N.Y., Lian J.Y., Wang P.F., Xu Z.B. (2022). Recent progress in minimizing the warpage and shrinkage deformations by the optimization of process parameters in plastic injection molding: A review. Int. J. Adv. Manuf. Technol..

[31] Guerra N.B., Reis T.M., Scopel T., de Lima M.S., Figueroa C.A., Michels A.F. (2023). Influence of process parameters and post-molding condition on shrinkage and warpage of injection-molded plastic parts with complex geometry. Int. J. Adv. Manuf. Technol..

[32] Wilczyński K., Wilczyński K.J., Buziak K. (2022). Modeling and experimental studies on polymer melting and flow in injection molding. Polymers.

[33] Dekel Z., Kenig S. (2021). Micro-injection molding of polymer nanocomposites composition-process-properties relationship. Int. Polym. Process..

[34] Baum M., Anders D., Reinicke T. (2023). Approaches for numerical modeling and simulation of the filling phase in injection molding: A Review. Polymers.

[35] Wang J., Mao Q., Jiang N., Chen J. (2021). Effects of injection molding parameters on properties of insert-injection molded polypropylene single-polymer composites. Polymers.

[36] Kuo C.C., Qiu S.X., Lee G.Y., Zhou J., He H.Q. (2021). Characterizations of polymer injection molding tools with conformal cooling channels fabricated by direct and indirect rapid tooling technologies. Int. J. Adv. Manuf. Technol..

[37] Fu H., Xu H., Liu Y., Yang Z., Kormakov S., Wu D., Sun J. (2020). Overview of injection molding technology for processing polymers and their composites. ES Mater. Manuf..

[38] Li K., Yan S., Zhong Y., Pan W., Zhao G. (2019). Multi-objective optimization of the fiber-reinforced composite injection molding process using Taguchi method, RSM and NSGA-II. Simul. Model. Pract. Theory.

[39] Amjadi M., Fatemi A. (2021). A fatigue damage model for life prediction of injection molded short glass fiber reinforced thermoplastic composites. Polymers.

[40] Lee J., Lee H., Kim N. (2023). Fiber orientation and strain rate-dependent tensile and compressive behavior of injection molded polyamide-6 reinforced with 20% short carbon fiber. Polymers.

[41] Ucpinar Durmaz B., Artykbaeva E., Aytac A. (2024). Fabrication and performance of short glass fiber reinforced polyamide composites. Int. J. Polym. Anal. Charact..

[42] Artykbaeva E., Ucpinar Durmaz B., Aksoy P., Aytac A. (2022). Investigation of the properties of PA6/PA610 blends and glass fiber reinforced PA6/PA610 composites. Polym. Compos..

[43] Tan F., Birişik B. (2025). Optimization of material and process parameters in the injection molding of piezoresistive card-type pressure sensors using the finite element method. J. Sci. Rep.-A.

[44] Tan F., Alkan A. (2024). Effect of Cooling Parameters on In-Mold Flow Behavior in the Microinjection Molding of Piezoelectric Pumps. Int. J. Automot. Sci. Technol..

[45] Tan F. (2020). Experimental Investigation of Mechanical Properties for Injection Molded PA66+PA6I/6T Composite Using RSM and Grey Wolf Optimization. El-Cezeri.

[46] Tan F., Alkan A. (2025). Numerıcal investigation of thermal and structural behavior in injection-molded femur implants. Kahramanmaras Sutcu Imam Univ. J. Eng. Sci..

[47] Advani S.G., Tucker C.L. (1987). The Use of Tensors to Describe and Predict Fiber Orientation in Short Fiber Composites. J. Rheol..

[48] Ryu Y., Sohn J.S., Yun C.-S., Cha S.W. (2020). Shrinkage and Warpage Minimization of Glass-Fiber-Reinforced Polyamide 6 Parts by Microcellular Foam Injection Molding. Polymers.

